# Atopic dermatitis phenotypes in childhood

**DOI:** 10.1186/1824-7288-40-46

**Published:** 2014-05-12

**Authors:** Giampaolo Ricci, Arianna Dondi, Iria Neri, Lorenza Ricci, Annalisa Patrizi, Andrea Pession

**Affiliations:** 1 Pediatric Unit, Department of Medical and Surgical Sciences, University of Bologna, Via Massarenti 9, 40138 Bologna, Italy; 2 Dermatology Unit, Department of Specialistic, Diagnostic and Experimental Medicine, Bologna, Italy

**Keywords:** Atopic dermatitis, Phenotypes, Allergy, Childhood, Atopic march

## Abstract

**Background:**

Atopic dermatitis (AD) is a chronic inflammatory skin disease and can be the first step of the atopic march.

**Objective:**

In this retrospective study, we analysed the immunological and clinical patterns of AD in a group of children affected by the disease since their first years of life, in order to evaluate if and how these patterns can change over time, and to identify biomarkers that can possibly correlate with the clinical phenotype.

**Methods:**

We enrolled Caucasian children with diagnosis of AD performed by a specialist on the basis of Hanifin and Rajka’s criteria and with a first clinical and laboratory evaluation before 5 years of age. Patients were divided in 2 groups: IgE-associated AD (with or without allergic respiratory diseases) and non-IgE-associated AD.

**Results:**

Among 184 patients enrolled in this study, at the beginning 30/184 were classified as having non-IgE-associated AD, but during follow-up, at the median age of 36 months, 15 patients became allergic. All 15 patients who switched from the non-IgE to the IgE-associated form had a significantly earlier onset of AD than those who did not switch. Dust mite sensitization seem to be the best biomarker (OR 2.86) to predict the appearance of allergic respiratory diseases.

**Conclusion:**

IgE-associated AD is more frequent in childhood than non-IgE-associated AD. These two phenotypes are different in the age of onset and in the remission patterns. In the first years of life, it is important to distinguish the different phenotypes in order to evaluate possible allergic related conditions.

## Introduction

Atopic dermatitis (AD) is a chronic inflammatory skin disease that affects between 15% to 30% of the pediatric population with differences according to age [1,2]. The presence of an epidermal barrier dysfunction and an alterated immunoallergic profile contribute to AD pathogenesis [3,4]. Filaggrin mutations, other genetically transmitted skin barrier defects, as well as environmental factors, form the basis for the clinical heterogeneity of the disease [5,6].

Food allergy is more common in children with AD than in the general population, being present in 15-30% of these patients [7-9]. Moreover AD, especially the early-onset kind, could represent the first step of the atopic march [10,11] and it is a major risk factor for the development of asthma; and patients with specific IgE antibodies to common environmental allergens present a higher risk for progressing in the atopic march to respiratory allergic diseases, such as rhinitis and asthma, than those without IgE sensitization. These diseases may appear subsequently to or more rarely simultaneously with AD [12,13]. The adaptive immune response in AD is associated with increased expression of the TH2 cytokines (IL-4, IL-13 and IL-31) during the acute phase of AD, while in the chronic phase also TH1 polarization is present [14].

Despite its high incidence, previous studies that investigated the different immunological parameters of AD, such as total and specific IgE (sIgE) serum levels, have shown a heterogeneity of results probably due to different patient selection criteria and to an inhomogeneous determination of the allergometric tests [15-24].

Several systemic and cutaneous immune abnormalities have been previously described in AD [25]. Among them, an abnormal Th2-polarization of the immune response [26] might play a pivotal role and has also been reported in association with a higher incidence of asthma and allergies.

Recently, a biological model has been proposed in order to try to identify the links between AD genotype and the endophenotype [27], and, possibly, to develop distinctive preventive measures and personalized therapeutic approaches [28]. A better understanding of the different AD phenotypes is very important to this respect [29].

The primary endpoint of this retrospective follow-up study is to analyze the immunological and clinical patterns of AD in a group of children affected by the disease since their first years of life. Other endpoints are 1) to evaluate if and how these patterns can change over time and 2) to identify the presence of biomarkers that can possibly correlate with the clinical phenotype.

## Materials and methods

### Study population and inclusion criteria

The present report represents the retrospective preliminary stage of a study named “AllerGene2” that aims at correlating AD phenotypes to genetic features, in particular IgE and non-IgE-associated forms.

We enrolled Caucasian children with AD referred to the Pediatric Dermatology (Dermatology Unit) and Pediatric Allergology (Pediatric Unit) Outpatient Clinics of S. Orsola-Malpighi University Hospital in Bologna from 2006 to 2011. Patients involved in this study had already been included in the normal clinical routine of these departments.

Patients were enrolled if the following inclusion criteria were fulfilled:

a) an informed consent signed by the parents;

b) an age between 0 and 18 years;

c) a first clinical and laboratory evaluation at ≤5 years of age;

d) a diagnosis of AD performed by a specialist of the team based on the criteria of Hanifin and Rajka [30];

e) a mean retrospective follow-up of 10 years.

Patients were excluded if they suffered from a systemic disease (other than asthma or allergy) or in the absence of parents’ consent.

All the included patients were required to have performed, either for AD evaluation or for food allergy, asthma and allergic rhinitis follow-up, a complete allergometric assessment defined by skin prick test (SPT) and total and s-IgE.

### Clinical assessment

At the time of the first evaluation, AD severity was assessed by means of the objective SCORAD [31]. We considered AD mild when the objective SCORAD was lower than 20 points, moderate between 20 and 39, severe 40 points or more.

At each visit, when clinically appropriate, allergometric tests were performed to define the AD phenotype: SPT for common inhalant and food allergens and/or total and sIgE serum level.

We considered AD as IgE-associated if at least one of the following criteria was observed:

1. clinical symptoms of allergic diseases (asthma and/or rhinoconjunctivitis and/or food allergy);

2. positive SPT for food or inhalant allergens;

3. elevated level of total IgE according to age [32];

4. presence of sensitization for food or inhalant allergens (sIgE measured by ImmunoCAP method).

On the contrary, we defined non-IgE-associated AD if none of the previous criteria was satisfied [33].

The group with IgE-associated AD was further divided into 2 subgroups: 1) patients with allergic respiratory symptoms and 2) patients without allergic respiratory symptoms. The mean follow-up time from AD diagnosis to inclusion in the present analysis was 10 years.

### Allergometric assessment

SPT (extracts by Lofarma, Italy) were performed for the most common food (cow’s milk, hen’s egg, soybean, wheat, peanut, nut, codfish, apple) and inhalant allergens (grass pollens; *D. pteronyssinus*; *D. farinae*; dog and cat dander; *Alternaria a.)* and for history-oriented allergens.

The total IgE serum level was determined with ELISA and was compared with the normal value for age [32].

The determination of sIgE was performed by ImmunoCAP™ FEIA (Phadia, Sweden) in all patients for the same allergens as above. A patient was considered as sensitized when having sIgE levels higher than 0.70 kU/L for food allergens, and higher than 0.35 kU/L for inhalant allergens. We assumed a different and higher cut-off value for food allergens because weak positive values of sIgE for the main foods (cow’s milk, hen’s egg, wheat) may occur in the first years of life even in normal subjects, according to the results of the Danish Allergy Research Center cohort [34].

### Statistical methods

Standard statistical descriptions of parameters were used to characterize the data (mean, median and range). The outcomes of persistence or remission of AD were evaluated in the different groups and graphically represented using the method of Kaplan and Meier. Student t test was performed to compare means between groups. All P values were 2-sided and values less than 0.05 were considered as statistically significant.

Statistics were performed using Microsoft Excel® version 2007 for Windows 7 and GraphPad QuickCalcs Online Calculators for Scientists [35].

### Ethical aspects

The AllerGene2 study was approved by the S. Orsola- Malpighi Hospital Ethics Commettee on 10^th^ May 2011 protocol n. 40/2011/U/Tess.

## Results

**Figure 1:**
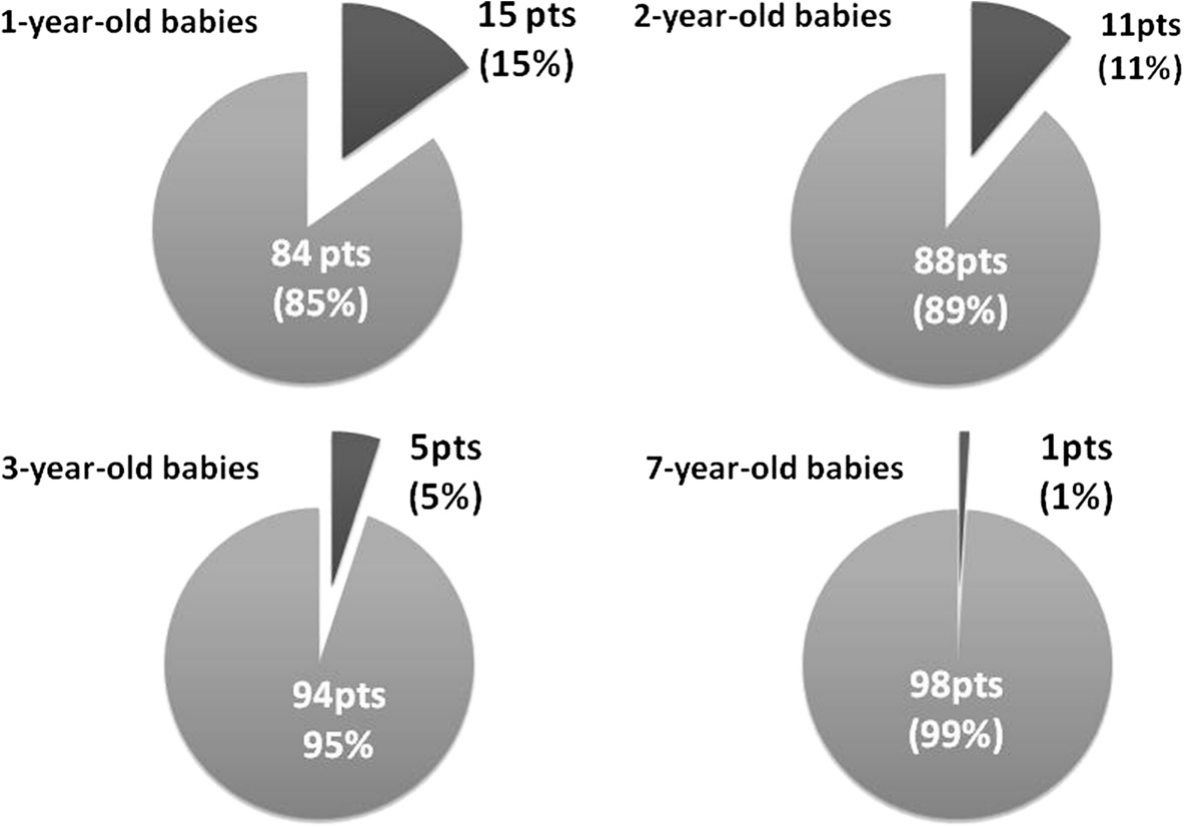
**Switch from non-IgE to IgE-associated AD in 99 children studied before 2 years of age.** The switch happened in all cases by the age of 8 years.

A total of 184 patients entered the study. At time 0, that is the first clinical and laboratory evaluation, 99 patients were aged under 2 years and 85 patients were between 2 and 5 years. At the first clinical evaluation 35 children (19%) had mild AD, 112 (61%) moderate AD and 37 (20%) severe AD.30/184 (16%) had been classified as having non-IgE-associated AD. Among these 30 non-IgE AD patients, at a median age of 36 months, 15 children became allergic, so that at time 1, after a mean follow-up of 10 years, of the total 184 patients, 169 (92%) were classified as having IgE-associated and 15 (8%) non-IgE-associated AD. All 15 patients who switched from the non-IgE- to the IgE-associated form had an early onset of AD and had been first referred to our Clinics before the age of 2; in particular, 4 children switched between the first and second year of life, 6 patients between the second and third year, 4 between the fourth and the seventh, and the last one before the eighth year (Figure 1).

**Figure 2:**
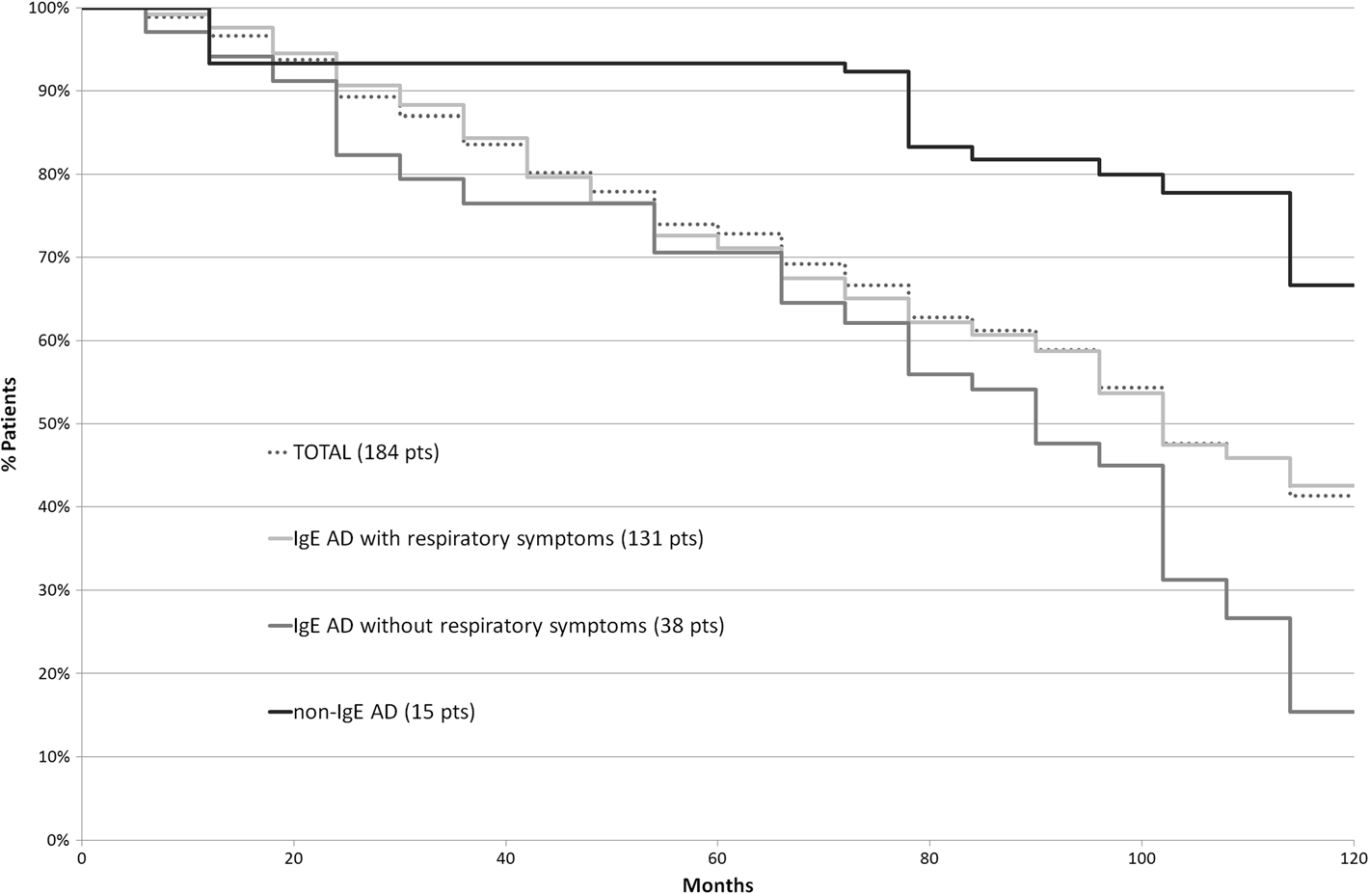
**AD remission over time (Kaplan-Meier graph).** “Total” represents all the included patients (N = 184); IgE-associated AD (N = 169) was divided between children with (N = 131) and without (N = 38) allergic respiratory diseases; non-IgE-associated AD (N = 15).

**Figure 3:**
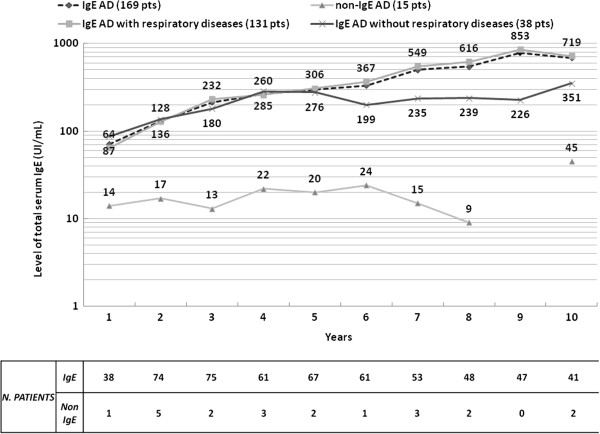
**Mean levels of total serum IgE (UI/mL) over time in the different groups.** Geometric means are plotted as logarithms.

At the end of the follow-up 169 (92%) were diagnosed with IgE-associated AD and 15 (8%) with the non-IgE-associated form; the main clinical characteristics are reported in Table 1. The mean age of onset and of remission of AD was significantly different between the 2 phenotypes: respectively 8 months vs 30 months (p = 0.0001) for onset, 61 months vs 119 months for remission (p = 0.0003).AD course was longer in children affected by non-IgE-associated AD: in particular, it persisted in more than 90% (14/15) of patients at 60 months of life and in about 67% (10/15) of patients at 120 months (10 years). In the IgE-associated form, remission occurred earlier (Figure 2); in those patients without respiratory symptoms, AD was present in roughly 70% of children at 60 months of life and in less than 20% of patients at 120 months.Moreover, the geometric mean values of total IgE increased year by year in IgE-associated AD, whereas in the non-IgE-associated form the total IgE level remained constantly low over time (Figure 3).

**Table 1:** Main clinical features of 184 children affected by AD after a mean 10-year follow-up

	**Non-IgE AD**	**IgE AD**	***IgE AD with respiratory diseases***	***IgE AD without respiratory diseases***	**Total**
Patients N (%)	15 (8%)	169 (92%)	*131 (78%)*	*38 (22%)*	184
M (%)	7 (47%)	95 (56%)	*74 (56%)*	*21 (55%)*	102 (55%)
Family history of atopy N (%)	10 (67%)	120 (71%)	*94 (72%)*	*26 (68%)*	130 (71%)
Family history of AD N (%)	1 (7%)	23 (14%)	*19 (15%)*	*4 (11%)*	24 (13%)
Mean age at AD onset (months)	30 *	8 *	*8*	*7*	10
Mean age of AD remission (months)	119 **	61 **	***65 ***^***$***^	***36 ***^***$***^	66
Past or present food allergy/OAS	/	125 (74%)	*94 (72%)*	*31 (82%)*	125 (68%)
Mean age at food allergy/OAS onset (months)	/	16	*18*	*9*	
Mean age at food allergy/OAS remission (months)	/	49	***55 ***^***$$***^	***37 ***^***$$***^	
Wheezing	/	53 (31%)	*53 (40%)*	*/*	53 (29%)
Asthma	/	62 (37%)	*62 (47%)*	*/*	62 (34%)
Allergic Rhinitis N (%)	/	112 (66%)	*112 (85%)*	*/*	112 (61%)

^*^P value = 0.0001; ^**^P value = 0.0003; ^$^P value = 0.0146; ^$$^ P value = 0.035.

IgE-associated AD (169 children) was divided in 2 subgroups: 1) with allergic respiratory symptoms (131 children); 2) without allergic respiratory symptoms (38 children). OAS = oral allergy syndrome.

The IgE-associated group was divided into 2 subgroups (Table 1):

a) group 1, with respiratory allergy (N 131, 78%), in which asthma was present in 62 patients (47%, mean age at onset 6 years) and rhinoconjunctivitis in 112 (86%, mean age at onset 6 years);

b) group 2, without respiratory allergy (N 38, 22%).

The mean age of AD remission was 65 months in group 1 and 36 months in group 2 (p = 0.0146); moreover the mean age of food allergy remission was 55 months in group 1 and 37 months in group 2 (p = 0.035) (Table 1).

The mean values of sIgE against the main food and inhalant allergens at different ages are reported in Table 2. Subjects with respiratory diseases showed higher levels of total and sIgE for inhalant allergens; in particular, dust mites were higher both in absolute value and in percentage, mainly in the first 5 years of life; children with inhalants sensitization in the first 3 years of life, have a higher risk to be affected by respiratory allergy in later years (dust mite sensitization OR 2.86; grass pollen OR 1.66).

**Table 2:** Geometric mean (GM) of total (UI/mL) and specific (kU/L) IgE, and percentage of positivity in children with IgE-associated AD phenotype at different ages

**IgE-mediated AD with respiratory diseases**														
**Months**	**0-12**		**13-24**		**24-36**		**36-48**		**48-60**		**61-72**		**73-84**	
	**(N = 25)**		**(N = 51)**		**(N = 49)**		**(N = 45)**		**(N = 53)**		**(N = 59)**		**(N = 47)**	
**IgE (UI/mL)**	64		128		232		260		306		367		549	
**sIgE (kU/L)**	*GM*	*%*	*GM*	*%*	*GM*	*%*	*GM*	*%*	*GM*	*%*	*GM*	*%*	*GM*	*%*
Cow’s milk	6.2	*48*	3.0	*63*	2.4	*69*	1.6	*53*	2.0	*42*	2.0	*37*	2.1	*38*
Hen’s egg (white)	7.3	*76*	5.8	*76*	4.8	*73*	3.5	*67*	2.6	*57*	1.7	*42*	2.4	*40*
*Phleum p.*	0.8	*12*	1.4	*14*	2.0	*47*	4.7	*69*	8.3	*72*	13.5	*63*	16.9	*87*
*Cynodon d.*	0	2.3	*6*	1.6	*38*	3.0	*51*	3.7	*60*	7.2	*49*	9.4	*81*	
*D. pteronyssinus*	1.1	*12*	1.5	*10*	4.0	*18*	5.5	*40*	8.0	*40*	9.1	39	11.0	*53*
*D. farinae*	0.5	*8*	1.1	*10*	2.6	*22*	6.9	*40*	8.5	*34*	7.3	*37*	13.7	*49*
**IgE-mediated AD without respiratory diseases**														
**Months**	**0-12**		**13-24**		**24-36**		**36-48**		**48-60**		**61-72**		**73-84**	
	**(N = 13)**		**(N = 25)**		**(N = 25)**		**(N = 16)**		**(N = 15)**		**(N = 11)**		**(N = 6)**	
**IgE (UI/mL)**	87		136		180		285		276		199		235	
**sIgE (kU/L)**	*GM*	*%*	*GM*	*%*	*GM*	*%*	*GM*	*%*	*GM*	*%*	*GM*	*%*	*GM*	*%*
Cow’s milk	3.7	*69*	6.6	*56*	5.1	*60*	5.2	*62*	1.9	*53*	9.5	*36*	52.0	*33*
Hen’s egg (white)	6.8	*92*	4.8	*72*	2.7	*56*	2.1	*62*	2.2	*73*	1.6	*45*	0.7	*33*
*Phleum p.*	0	0.5	*12*	3.1	*24*	6.2	*50*	4.9	*47*	24.8	*54*	13.8	*33*	
*Cynodon d.*	0	0.5	*16*	1.9	*20*	3.5	*50*	4.7	*40*	10.1	*54*	4.4	*33*	
*D. pteronyssinus*	0	0.4	*4*	0.6	*8*	1.1	*25*	1.7	*33*	3.9	*36*	8.1	*33*	
*D. farinae*	0	0.4	*4*	1.1	*8*	1.6	*19*	1.7	*33*	4.0	*36*	5.6	*33*	

The patients are divided into 2 groups according to the presence (group 1) or absence (group 2) of allergic respiratory diseases. Total and specific IgE were periodically performed according to the patients’ needs.

The results of SPT did not influence the definition of the patient’s phenotype; i.e. all patients with at least one positive SPT had at least one positive sIgE, and all the patients with negative SPT had negative sIgE.

## Discussion

Although many clinical and laboratory studies have tried to understand AD and the progression of the atopic march, few data exist in order to determine the different phenotypes of AD. In a previous review, Schmid-Grendelmeier et al. [15]. reported the presence of many differences between non-IgE-associated and IgE-associated AD: in particular, data about the prevalence of the non-IgE-associated form in the pediatric age are conflicting, ranging from 18% in the paper of Kalinke et al. [16] who studied 22 children aged 1–5 years, to 45% observed in the paper of Cabon et al. [20] in 59 children aged 0–12 years. In 1999, Schäefer et al. [23]. reported 25% of non-IgE-associated AD forms in an epidemiological study on 2201 children aged 5–14 years.

On the other hand, Palmer et al. [24]. observed that around 80% of infants with AD exhibit increased total serum IgE levels.

Our data support the concept of the high frequency of IgE-associated AD in children (N. 169/184, 92%). This value can, however, be partially biased by the preliminary selection of the patients that are normally referred to us. Children with mild AD are rarely referred to a third-level allergological or dermatological clinic, because they are usually treated by the primary care pediatrics and only the more severe or persistent forms need a deeper clinical, dermatological and allergological evaluation. This might explain the fact that only a fifth of our children presented a mild form of AD: the majority of them had moderate or severe forms, in which an allergic background coexists very frequently, mainly when the child is affected by recurrent wheezing.

An interesting point raised by our study is that patients without an allergic sensitization should be considered as having a non-IgE-associated form around school-age and not earlier: most of the 15 children who passed from a non-IgE-associated to an IgE-associated form switched in the first 3 years of life and all of them within the seventh year (Figure 1). Other authors [36,37] have previously mentioned the possibility of a switch from non-IgE- to IgE-associated AD, especially in the first years of life and only rarely afterwards.

Some clinical characteristics of non-IgE-associated AD reported in this study are similar to those reported in previous studies: [15,38-42] for example, the later onset and the delayed remission; whereas there is no significant difference in the family history compared to IgE-associated AD.

According to our data, in order to define the form of AD, it might be sufficient to determine only one parameter between sIgE and SPT, excluding total IgE [43]: certainly, sIgE are more sensitive, in particular for inhalant allergens in the first years of life, but in our study when only SPT were performed, the patients distribution between IgE and non-IgE-associated AD did not change, because of multiple inhalant and/or food sensitizations in the majority of children.

All the patients who switched from non IgE- to IgE-associated AD forms were studied in the first 2 years of life. We feel it important to emphasize that IgE-associated AD has an early onset age (mean age 8 months vs 30 months of non-IgE-associated form), supporting the hypothesis that the allergen may penetrate through the inflamed skin of the child and initiate the allergic response [3,44].

A complete remission of skin symptoms (in about 50% of patients) was observed around 6–7 years of age in the whole group, confirming the previous data by Einchenfield et al. [45]. that reported between 40-60% of AD remission at the age of 7.

We observed that in patients with IgE-associated AD, total and sIgE levels grow steadily with age, for the development of respiratory and/or food allergies, whereas in the other form, total IgE remain low and stable over time (Figure 3 and Table 2).Furthermore, because the IgE-associated AD group was much larger, we attempted to define another clinical phenotype by correlating the presence of respiratory allergy with other possible/probable biomarkers. We distinguished patients with allergic respiratory diseases (group 1) from those without allergic respiratory diseases (group 2): at the same age of follow-up, even though AD remission cases are overlapping, the mean age of AD remission is statistically lower in the subgroup without respiratory allergies (36 months vs 65 months, P = 0.0146) (Figure 2); a possible explanation for this difference might be that during the pollen season, in the presence of rhinitis or asthma, there could be a worsening of AD.

Patients with allergic respiratory diseases show a higher concentration and percentage of sIgE for inhalant allergens than those in group 2, with sensitizations only against inhalants (Table 2). These early sensitizations to grass pollens and mainly to mites can represent a significant biomarker, predicting the appearance of respiratory diseases, especially asthma.

The onset of food allergy in the first years of life is related to the presence of cow’s milk and/or hen’s egg sensitization, as confirmed by high sIgE levels for these allergens in both groups (Table 2); later, in the subgroup with respiratory diseases, most cases of immediate symptoms after food ingestion are due to oral allergy syndrome to fruits and vegetables which include allergens that cross-react with pollens (data not shown).

Clinical AD phenotypes evolve during the first years of life so that it is difficult to frame each patient into a definite classification until a sufficient follow-up period has been reached. It is not yet possible to predict if the patients with only food allergy symptoms, but sensitized to inhalant allergens, will also develop respiratory symptoms in the following years, as well as we cannot predict if a patient with only allergic rhinitis and high levels of sIgE will also develop asthma. In line with what was suggested by Bieber et al. [27],. the correlation with different genotypes will maybe suggest some additional data in order to understand the complexity of the mechanisms underlying the different clinical phenotypes and possibly link them to specific biomarkers.

A bias of our study is that some patients with mild AD were not followed up for a sufficient time and were not included in this study, so that our conclusions are especially addressed to moderate to severe AD.

Our study shows that IgE-associated AD is more frequent in childhood than non-IgE associated AD and that these two phenotypes are different in the age of onset, which is usually later in the case of the non-IgE forms, and in the remission patterns, since IgE-associated AD seems to remit earlier and more frequently than the other form.

In the first 3 years of life, it is useful to evaluate the atopic condition in moderate-to-severe AD, and SPT are often sufficient to distinguish between the IgE- and non IgE-associated forms. In particular, it is important to pay attention to the sensitization for dust mites in the first 3 years of life, because it is a possible marker for subsequent appearance of allergic respiratory diseases.

## Abbreviations

AD: Atopic dermatitis; sIgE: Specific Immunoglobulin E; TH2: T-Helper 2 lymphocytes; SPT: Skin prick test; SCORAD: Scoring of atopic dermatitis; ELISA: Enzyme-linked immunosorbent assay; OR: Odds ratio; OAS: Oral allergy syndrome; GM: Geometric mean.

## Competing interests

Authors declare no conflict of interest.

## Author’s information

Annalisa Patrizi and Andrea Pession are co-seniorship.

## Authors’ contributions

GR conception and design of the study, data acquisition, analysis and interpretation, approval of the final version; AD conception and design of the study, data analysis and interpretation, manuscript drafting and revision, approval of the final version; IN conception and design of the study and manuscript revision, approval of the final version; LR data acquisition, analysis and interpretation, manuscript drafting and revision, approval of the final version; APa manuscript revision, approval of the final version; APe manuscript revision, approval of the final version.
